# Potential Role of Free Fatty Acids in the Pathogenesis of Periodontitis and Primary Sjögren’s Syndrome

**DOI:** 10.3390/ijms18040836

**Published:** 2017-04-14

**Authors:** Yosuke Shikama, Yasusei Kudo, Naozumi Ishimaru, Makoto Funaki

**Affiliations:** 1 Department of Oral Disease Research, National Center for Geriatrics and Gerontology, 7-430 Morioka-cho, Obu 474-8511, Japan; 2 Department of Oral Molecular Pathology, Tokushima University Graduate School, 3-18-15 Kuramoto-cho, Tokushima 770-8504, Japan; yasusei@tokushima-u.ac.jp (Y.K.); ishimaru.n@tokushima-u.ac.jp (N.I.); 3 Clinical Research Center for Diabetes, Tokushima University Hospital, 2-50-1 Kuramoto-cho, Tokushima 770-8503, Japan; m-funaki@tokushima-u.ac.jp

**Keywords:** periodontitis, Sjögren’s syndrome, free fatty acid, type 2 diabetes, metabolic disorder

## Abstract

Clinical studies have shown that metabolic disorders such as type 2 diabetes and dyslipidemia are associated with increased risk of oral-related diseases, such as periodontitis and Sjögren’s syndrome. Although changes in the immune system are critical in both of these metabolic disorders and oral-related diseases, the mechanism underlying the interaction between these diseases remains largely unknown. Obesity and type 2 diabetes are known to be associated with higher concentrations of free fatty acids in blood. Among free fatty acids, saturated fatty acids such as palmitic acid have been demonstrated to induce inflammatory responses mainly via the innate immune systems, and to be involved in the pathogenesis of type 2 diabetes in tissues such as adipose tissue, liver, pancreas, and skeletal muscle. Here, we highlight recent advances in evidence for the potential involvement of palmitic acid in the pathogenesis of periodontitis and Sjögren’s syndrome, and discuss the possibility that improvement of the lipid profile could be a new strategy for the treatment of these diseases.

## 1. Introduction

Obesity is a global health issue which is related to morbidity and mortality of metabolic diseases. Obese people have a more than ten-fold risk of developing type 2 diabetes (T2D) compared with normal-weight people [1]. The level of free fatty acids (FFAs) in blood is elevated in obese individuals and patients with T2D, as well as in animal models of these conditions [2], and is related to augmented lipolysis in adipocytes and an increased intake of dietary fats [3]. Potential intracellular mechanisms whereby FFAs cause insulin resistance have been explored, and a role of several inflammatory signaling networks has emerged. Intracellular kinases linked to inflammatory signaling, such as protein kinase C (PKC)-θ, IkB kinase (IKK) α, and c-jun N-terminal kinase (JNK) appear to play roles in FFA-induced insulin resistance [4,5,6,7]. Plasma contains a variety of long-chain FFAs, of which about 35% are saturated and 65% are unsaturated [8]. Among FFAs, saturated fatty acids, such as palmitic acid (Pal) and stearic acid, induce inflammatory responses mainly via the Toll-like receptor (TLR) signaling pathway [9,10]. Furthermore, fatty acid translocase, which is also known as CD36, is involved in FFA uptake [11], and CD36 ligands facilitate sterile inflammation through assembly of TLR heterodimers [12].

Periodontitis is a chronic bacterial infection that stimulates a host inflammatory response, leading to periodontal tissue damage [13]. Clinical studies have demonstrated that obesity, diabetes, and metabolic syndrome are associated with an increased risk of periodontitis [14,15,16], suggesting that lipid-related disorders, which are present in these diseases, may increase the risk of developing periodontitis. However, the molecular mechanisms underlying the association between disorders of lipid metabolism and periodontitis remain largely unknown.

Primary Sjögren’s syndrome (SS) is an autoimmune condition characterized by progressive lymphocytic infiltration of the salivary and lacrimal glands. It is known that salivary gland epithelial cells have an active role in the inflammatory process of SS [17]. The relationship between metabolic disorders and SS was firstly demonstrated as “pseudo-SS” [18]. Subsequent studies reported that patients with primary SS exhibited a markedly higher prevalence of metabolic disorders, such as diabetes and dyslipidemia [19,20]. However, the clinical significance of FFAs in the pathogenesis of SS is still unclear.

Herein, we introduce the potential involvement of FFAs, especially Pal, in the pathogenesis of periodontitis and SS, and discuss whether improvement of the lipid profile may be a new strategy for treating these diseases.

## 2. Involvement of Pal in Pathogenesis of Periodontitis

Periodontitis is known as the sixth complication of diabetes [21]; diabetes has been found to be an important host risk factor for periodontal disease in large epidemiological studies [22,23]. Studies relating periodontitis to T2D, such as the Pima Indian study, have shown increased prevalence and incidence of periodontal disease in patients with diabetes [24]. Experimental studies in animal models have also shown the influence of diabetes on periodontitis. In mice [25,26] and rats [27], a diabetic condition significantly increased the prevalence and severity of alveolar bone resorption in periodontitis induced by ligatures or bacterial infection. The effects of obesity on periodontitis have also been reported in animals and humans. In animals with periodontitis, greater alveolar bone loss was observed in obese mice [28] and rats [29] than in non-obese animals. In 1998, a relationship between obesity and periodontitis was first demonstrated in humans [30].

The level of FFAs in blood is increased in obese individuals [31], in patients with T2D, and in rodent models of T2D [2]. Recently, we demonstrated that: (i) Human gingival fibroblasts (HGF) express cell surface CD36 protein; (ii) CD36 expression was upregulated in gingival fibroblasts of diet-induced T2D model mice; (iii) Pal increased mRNA expression and secretion of interleukin (IL)-6, IL-8, and GROα, which are involved in host defense against periodontal lesions [32], in HGF; (iv) Saturated fatty acids, but not an unsaturated fatty acid, oleic acid, stimulated IL-8 secretion; (v) An omega-3 polyunsaturated fatty acid, docosahexaenoic acid (DHA), markedly decreased Pal-induced IL-6 and IL-8 secretion in HGF; (vi) Sulfosuccimidyl oleate sodium, a CD36 inhibitor, also suppressed Pal-induced pro-inflammatory responses in HGF; and (vii) Lipopolysaccharide and heat-killed component of *Porphyromonas gingivalis* (*P.g.*), which is an important periodontopathogen [33], augmented Pal-induced chemokine production in HGF [34]. Moreover, interesting papers recently reported that: (i) Contrary to oleic acid (one of the monounsaturated fatty acids), Pal demonstrated inflammatory potential that could accelerate alveolar bone loss in experimental periodontal disease in obese mice and affect the pro-inflammatory osteoclastic response to *P.g.* infection in vitro [35]; and (ii) LPS derived from *Aggregatibacter actinomycetemcomitans*, which is another important periodontopathogen, augmented high-fat diet-induced CD36 expression in periodontal tissue [36]. In addition to their role in the pathogenesis of periodontitis, it is also reported that *P.g.* and *P.g.* LPS augment high-fat diet- and Pal-induced endothelial injury [37] and steatohepatitis [38]. Furthermore, we recently reported that Pal-stimulated monocytes up-regulate adhesion molecules in vascular endothelial cells [39]. This could further enhance migration of monocytes and neutrophils, which also plays an active role in pro-inflammatory responses in periodontal lesions [40,41].

**Figure 1:**
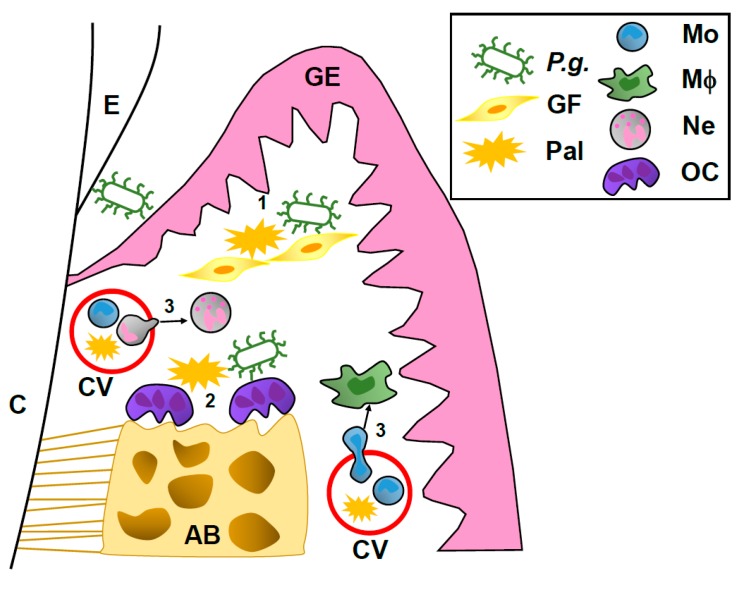
Proposed model of influence of Pal on pathogenesis of periodontitis. AB: alveolar bone, C: cementum, CV: capillary vessel, E: enamel, GE: gingival epithelium, GF: gingival fibroblast, Mo: monocyte, Mφ: macrophage, Ne: neutrophil, OC: osteoclast.

Considering these results, a proposed mechanism underlying the possible link between Pal in blood and the onset of periodontitis is shown in Figure 1. (1) Elevated Pal levels in blood may induce cytokine and chemokine secretion, and may augment *P.g.*-induced chemokine production in gingival fibroblasts, which promotes pro-inflammatory responses in periodontal lesions. (2) A high Pal level in plasma may augment *P.g.*-induced alveolar bone loss in human periodontal lesions. (3) Upregulation of adhesion molecules in vascular endothelial cells by Pal further enhances migration of monocytes and neutrophils, which also induces pro-inflammatory responses in periodontal lesions. This hypothesis suggests that a high level of Pal in plasma may be directly and indirectly involved in the pathogenesis of periodontitis.

## 3. Involvement of Pal in Pathogenesis of SS

Obesity, defined as body mass index above 30 kg/m^2^, cannot be considered only as an overweight condition with excessive fatty storage, but as a complicated state that exerts biological stress on many tissues and systems, including the immune system. Obesity seems to be a major environmental factor involved in the onset and progression of autoimmune disorders, including not only SS, but also rheumatoid arthritis, multiple sclerosis, psoriasis and psoriatic arthritis [42]. Moreover, as described in the Introduction, patients with primary SS exhibit a significantly higher prevalence of metabolic disorders, such as diabetes and dyslipidemia [19,20]. Thus, it could be reasonable to hypothesize the involvement of FFAs, especially Pal, in the onset/progression of SS, which is further discussed below.

**Figure 2:**
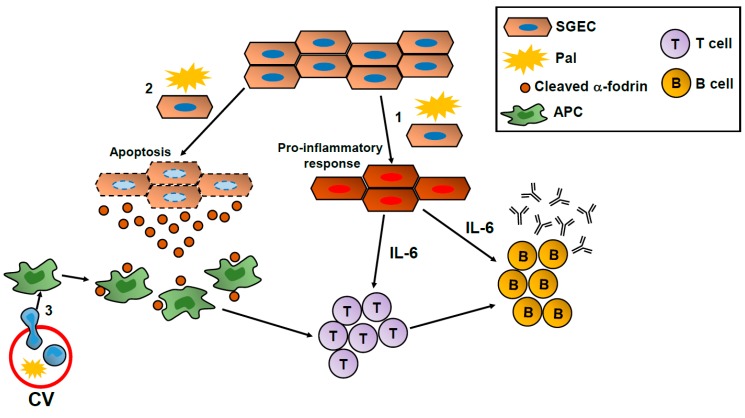
Potential mechanism of involvement of Pal in the pathogenesis of SS. APC: antigen presenting cell, CV: capillary vessel, SGEC: salivary gland epithelial cell.

It is known that salivary gland epithelial cells play an important role as a trigger in the development of SS. For example, IL-6 is upregulated in ductal epithelial cells of salivary glands in patients with primary SS. Furthermore, the extent and intensity of IL-6 expression in epithelial cells correlated with the grade of mononuclear cell infiltration [43]. α-fodrin is a ubiquitous, heterodimeric calmodulin-binding protein that is cleaved during apoptosis by caspase-3 or μ-calpain. Besides the ribonucleoprotein particles SS-A/Ro and SS-B/La [44], these 120 kDa fragments derived from α-fodrin have been demonstrated to act as auto-antigens in patients with primary SS [45]. Although the molecular mechanisms underlying the relationship between metabolic disorders and SS are largely unclear, we previously demonstrated that Pal induces IL-6 secretion and α-fodrin cleavage in salivary gland epithelial cell lines, suggesting a possible link between the pathogenesis of primary SS and Pal levels in blood [46]. When model mice for primary SS [47] were fed a high-fat diet, their salivary glands and lacrimal glands exhibited inflammation significantly more advanced than that observed in model mice fed a normal diet. Moreover, although a preliminary finding, auto-antibody concentrations in plasma were significantly increased in primary SS model mice fed a high-fat diet compared with those in model mice fed a normal diet. Given these results, a schematic model of the potential involvement of Pal in the pathogenesis of SS is shown in Figure 2. (1) Pal may induce IL-6 production in epithelial cells of these glands, which would augment local inflammation in salivary glands by directing the differentiation of IL-4-producing CD4+ T (T helper type 2) cells [48], inducing the maturation of B cells into antibody-secreting cells, and promoting the survival and maintenance of long-lived plasma cells [49,50,51]. (2) A high level of Pal in plasma may induce apoptosis in epithelial cells of salivary glands in vivo, resulting in cleaved α-fodrin release, which antigen-presenting cells such as macrophages and dendritic cells would recognize as an auto-antigen. (3) As described above, Pal induces adhesion molecules in vascular endothelial cells via IL-1 signaling involving monocytes [39], which could enhance monocyte migration to inflammatory lesions in the salivary glands of patients with primary SS.

## 4. Is Improvement of the Lipid Profile Effective for the Treatment of Periodontitis and SS?

Normalizing FFA levels has been proposed as a novel therapeutic approach for obesity and metabolic diseases [31,52]. Recent studies have demonstrated that lipid-related molecules could improve the condition of patients with periodontitis. For example, docosahexaenoic acid (DHA) supplementation improved the periodontal condition in patients with periodontitis [53], and resolvin D1, which is a derivative of DHA, decreased *P.g.*-induced chemokine secretion in HGF [54]. Moreover, resolvin E1, which is another type of lipid mediator derived from eicosapentaenoic acid (EPA), protects against local inflammation and osteoclast-mediated bone destruction in periodontitis [55]. We also confirmed that DHA markedly inhibited Pal-induced IL-6 and IL-8 production in HGF [34], presumably via the suppressive effect of DHA on nuclear factor-κB (NF-κB) activation [56] and TLR dimerization [10]. Considering that DHA and EPA supplementation does not induce a significant change in the percentage of Pal in total fatty acids in plasma phospholipids [57], the ratio of ω-3 polyunsaturated fatty acids to Pal in plasma may be an important factor in the improving effects of DHA, EPA, and these derivatives on the clinical condition in periodontitis. Supporting this hypothesis, it was reported that the ratio of n3 (anti-inflammatory)- to n6 (pro-inflammatory)-polyunsaturated fatty acids, namely (DHA + EPA)/arachidonic acid, is significantly lower in the gingival crevicular fluid of aggressive periodontitis patients than in healthy controls [58].

Some papers have reported beneficial effects of lipid-related molecules on the salivary glands both in vivo and in vitro. Leigh et al. [59] reported that a resolvin D1 biosynthetic pathway exists in murine and human salivary gland cells, and the distribution of resolvin D1 biosynthesis-related mediators is different in human salivary gland cells of healthy subjects and patients with SS, which could suggest that resolvin D1 is being produced but not delivered to target cells in the salivary glands of patients with SS. Resolvin D1 also blocks inflammation mediated by tumor necrosis factor-α (TNF-α), which is an inflammatory cytokine inducing apoptosis in salivary gland cells [60], and increases barrier function and cell polarity of salivary gland cells [61,62]. These reports imply that DHA supplementation may have preventive and therapeutic effects on inflammatory diseases of the salivary glands such as SS.

## 5. Conclusions

The pathogenesis and mechanisms of periodontitis and SS are highly complex, and many patients develop refractory disease. Although the development of these diseases does not always lead to death, quality of life in patients with these diseases is considerably decreased. Further research on the association between lipid-related molecules and the pathogenesis of these diseases is warranted in order to develop novel therapeutic strategies.

## References

[1] Kopelman P. (2007). Health risks associated with overweight and obesity. Obes. Rev..

[2] Boden G. (2002). Interaction between free fatty acids and glucose metabolism. Curr. Opin. Clin. Nutr. Metab. Care.

[3] Cnop M. (2008). Fatty acids and glucolipotoxicity in the pathogenesis of type 2 diabetes. Biochem. Soc. Trans..

[4] Hirosumi J., Tuncman G., Chang L., Gorgun C.Z., Uysal K.T., Maeda K., Karin M., Hotamisligil G.S. (2002). A central role for jnk in obesity and insulin resistance. Nature.

[5] Kim J.K., Fillmore J.J., Sunshine M.J., Albrecht B., Higashimori T., Kim D.W., Liu Z.X., Soos T.J., Cline G.W., O’Brien W.R. (2004). PKC-θ knockout mice are protected from fat-induced insulin resistance. J. Clin. Investig..

[6] Kim J.K., Kim Y.J., Fillmore J.J., Chen Y., Moore I., Lee J., Yuan M., Li Z.W., Karin M., Perret P. (2001). Prevention of fat-induced insulin resistance by salicylate. J. Clin. Investig..

[7] Yuan M., Konstantopoulos N., Lee J., Hansen L., Li Z.W., Karin M., Shoelson S.E. (2001). Reversal of obesity- and diet-induced insulin resistance with salicylates or targeted disruption of ikkbeta. Science.

[8] Watt M.J., Hoy A.J., Muoio D.M., Coleman R.A. (2012). Distinct roles of specific fatty acids in cellular processes: Implications for interpreting and reporting experiments. Am. J. Physiol. Endocrinol. Metab..

[9] Maloney E., Sweet I.R., Hockenbery D.M., Pham M., Rizzo N.O., Tateya S., Handa P., Schwartz M.W., Kim F. (2009). Activation of NF-κB by palmitate in endothelial cells: A key role for nadph oxidase-derived superoxide in response to tlr4 activation. Arterioscler. Thromb. Vasc. Biol..

[10] Snodgrass R.G., Huang S., Choi I.W., Rutledge J.C., Hwang D.H. (2013). Inflammasome-mediated secretion of IL-1β in human monocytes through TLR2 activation; modulation by dietary fatty acids. J. Immunol..

[11] Campbell S.E., Tandon N.N., Woldegiorgis G., Luiken J.J., Glatz J.F., Bonen A. (2004). A novel function for fatty acid translocase (fat)/CD36: Involvement in long chain fatty acid transfer into the mitochondria. J. Biol. Chem..

[12] Stewart C.R., Stuart L.M., Wilkinson K., van Gils J.M., Deng J., Halle A., Rayner K.J., Boyer L., Zhong R., Frazier W.A. (2010). CD36 ligands promote sterile inflammation through assembly of a toll-like receptor 4 and 6 heterodimer. Nat. Immunol..

[13] Darveau R.P. (2010). Periodontitis: A polymicrobial disruption of host homeostasis. Nat. Rev. Microbiol..

[14] Lalla E., Lamster I.B., Drury S., Fu C., Schmidt A.M. (2000). Hyperglycemia, glycoxidation and receptor for advanced glycation endproducts: Potential mechanisms underlying diabetic complications, including diabetes-associated periodontitis. Periodontol. 2000.

[15] Nibali L., Tatarakis N., Needleman I., Tu Y.K., D’Aiuto F., Rizzo M., Donos N. (2013). Clinical review: Association between metabolic syndrome and periodontitis: A systematic review and meta-analysis. J. Clin. Endocrinol. Metab..

[16] Suvan J., D’Aiuto F., Moles D.R., Petrie A., Donos N. (2011). Association between overweight/obesity and periodontitis in adults. A systematic review. Obes. Rev..

[17] Manoussakis M.N., Kapsogeorgou E.K. (2007). The role of epithelial cells in the pathogenesis of sjogren’s syndrome. Clin. Rev. Allergy Immunol..

[18] Goldman J.A., Julian E.H. (1977). Pseudo-sjogren syndrome with hyperlipoproteinemia. JAMA.

[19] Kang J.H., Lin H.C. (2010). Comorbidities in patients with primary sjogren’s syndrome: A registry-based case-control study. J. Rheumatol..

[20] Ramos-Casals M., Brito-Zeron P., Siso A., Vargas A., Ros E., Bove A., Belenguer R., Plaza J., Benavent J., Font J. (2007). High prevalence of serum metabolic alterations in primary sjogren’s syndrome: Influence on clinical and immunological expression. J. Rheumatol..

[21] Loe H. (1993). Periodontal disease: The sixth complication of diabetes mellitus. Diabetes Care.

[22] Loe H., Anerud A., Boysen H., Smith M. (1978). The natural history of periodontal disease in man. Tooth mortality rates before 40 years of age. J. Periodontal. Res..

[23] Loe H., Anerud A., Boysen H., Smith M. (1978). The natural history of periodontal disease in man: The rate of periodontal destruction before 40 years of age. J. Periodontol..

[24] Nelson R.G., Shlossman M., Budding L.M., Pettitt D.J., Saad M.F., Genco R.J., Knowler W.C. (1990). Periodontal disease and niddm in pima indians. Diabetes Care.

[25] Lalla E., Lamster I.B., Feit M., Huang L., Schmidt A.M. (1998). A murine model of accelerated periodontal disease in diabetes. J. Periodontal. Res..

[26] Lalla E., Lamster I.B., Feit M., Huang L., Spessot A., Qu W., Kislinger T., Lu Y., Stern D.M., Schmidt A.M. (2000). Blockade of rage suppresses periodontitis-associated bone loss in diabetic mice. J. Clin. Investig..

[27] Holzhausen M., Garcia D.F., Pepato M.T., Marcantonio E. (2004). The influence of short-term diabetes mellitus and insulin therapy on alveolar bone loss in rats. J. Periodontal. Res..

[28] Amar S., Zhou Q., Shaik-Dasthagirisaheb Y., Leeman S. (2007). Diet-induced obesity in mice causes changes in immune responses and bone loss manifested by bacterial challenge. Proc. Natl. Acad. Sci. USA.

[29] Perlstein M.I., Bissada N.F. (1977). Influence of obesity and hypertension on the severity of periodontitis in rats. Oral Surg. Oral. Med. Oral Pathol..

[30] Saito T., Shimazaki Y., Sakamoto M. (1998). Obesity and periodontitis. N. Engl. J. Med..

[31] Boden G., Shulman G.I. (2002). Free fatty acids in obesity and type 2 diabetes: Defining their role in the development of insulin resistance and β-cell dysfunction. Eur. J. Clin. Investig..

[32] Almasri A., Wisithphrom K., Windsor L.J., Olson B. (2007). Nicotine and lipopolysaccharide affect cytokine expression from gingival fibroblasts. J. Periodontol..

[33] Holt S.C., Ebersole J., Felton J., Brunsvold M., Kornman K.S. (1988). Implantation of bacteroides gingivalis in nonhuman primates initiates progression of periodontitis. Science.

[34] Shikama Y., Kudo Y., Ishimaru N., Funaki M. (2015). Possible involvement of palmitate in pathogenesis of periodontitis. J. Cell. Physiol..

[35] Muluke M., Gold T., Kiefhaber K., Al-Sahli A., Celenti R., Jiang H., Cremers S., Van Dyke T., Schulze-Spate U. (2016). Diet-induced obesity and its differential impact on periodontal bone loss. J. Dent. Res..

[36] Lu Z., Li Y., Brinson C.W., Kirkwood K.L., Lopes-Virella M.F., Huang Y. (2017). CD36 is upregulated in mice with periodontitis and metabolic syndrome and involved in macrophage gene upregulation by palmitate. Oral Dis..

[37] Ao M., Miyauchi M., Inubushi T., Kitagawa M., Furusho H., Ando T., Ayuningtyas N.F., Nagasaki A., Ishihara K., Tahara H. (2014). Infection with porphyromonas gingivalis exacerbates endothelial injury in obese mice. PLoS ONE.

[38] Furusho H., Miyauchi M., Hyogo H., Inubushi T., Ao M., Ouhara K., Hisatune J., Kurihara H., Sugai M., Hayes C.N. (2013). Dental infection of porphyromonas gingivalis exacerbates high fat diet-induced steatohepatitis in mice. J. Gastroenterol..

[39] Shikama Y., Aki N., Hata A., Nishimura M., Oyadomari S., Funaki M. (2015). Palmitate-stimulated monocytes induce adhesion molecule expression in endothelial cells via IL-1 signaling pathway. J. Cell. Physiol..

[40] Charon J., Toto P.D., Gargiulo A.W. (1981). Activated macrophages in human periodontitis. J. Periodontol..

[41] Ling M.R., Chapple I.L., Matthews J.B. (2015). Peripheral blood neutrophil cytokine hyper-reactivity in chronic periodontitis. Innate Immun..

[42] Versini M., Jeandel P.Y., Rosenthal E., Shoenfeld Y. (2014). Obesity in autoimmune diseases: Not a passive bystander. Autoimmun. Rev..

[43] Sekiguchi M., Iwasaki T., Kitano M., Kuno H., Hashimoto N., Kawahito Y., Azuma M., Hla T., Sano H. (2008). Role of sphingosine 1-phosphate in the pathogenesis of sjogren’s syndrome. J. Immunol..

[44] Chan E.K., Hamel J.C., Buyon J.P., Tan E.M. (1991). Molecular definition and sequence motifs of the 52-Kd component of human SS-A/RO autoantigen. J. Clin. Investig..

[45] Haneji N., Nakamura T., Takio K., Yanagi K., Higashiyama H., Saito I., Noji S., Sugino H., Hayashi Y. (1997). Identification of α-fodrin as a candidate autoantigen in primary sjogren’s syndrome. Science.

[46] Shikama Y., Ishimaru N., Kudo Y., Bando Y., Aki N., Hayashi Y., Funaki M. (2013). Effects of free fatty acids on human salivary gland epithelial cells. J. Dent. Res..

[47] Haneji N., Hamano H., Yanagi K., Hayashi Y. (1994). A new animal model for primary sjogren’s syndrome in NFS/SLD mutant mice. J. Immunol..

[48] Rincon M., Anguita J., Nakamura T., Fikrig E., Flavell R.A. (1997). Interleukin (IL)-6 directs the differentiation of IL-4-producing CD4+ T cells. J. Exp. Med..

[49] Jego G., Bataille R., Pellat-Deceunynck C. (2001). Interleukin-6 is a growth factor for nonmalignant human plasmablasts. Blood.

[50] Jego G., Palucka A.K., Blanck J.P., Chalouni C., Pascual V., Banchereau J. (2003). Plasmacytoid dendritic cells induce plasma cell differentiation through type i interferon and interleukin 6. Immunity.

[51] Rousset F., Garcia E., Banchereau J. (1991). Cytokine-induced proliferation and immunoglobulin production of human b lymphocytes triggered through their cd40 antigen. J. Exp. Med..

[52] Kusunoki J., Kanatani A., Moller D.E. (2006). Modulation of fatty acid metabolism as a potential approach to the treatment of obesity and the metabolic syndrome. Endocrine.

[53] Naqvi A.Z., Hasturk H., Mu L., Phillips R.S., Davis R.B., Halem S., Campos H., Goodson J.M., Van Dyke T.E., Mukamal K.J. (2014). Docosahexaenoic acid and periodontitis in adults: A randomized controlled trial. J. Dent. Res..

[54] Khaled M., Shibani N.A., Labban N., Batarseh G., Song F., Ruby J., Windsor L.J. (2013). Effects of resolvin d1 on cell survival and cytokine expression of human gingival fibroblasts. J. Periodontol..

[55] Hasturk H., Kantarci A., Ohira T., Arita M., Ebrahimi N., Chiang N., Petasis N.A., Levy B.D., Serhan C.N., Van Dyke T.E. (2006). Rve1 protects from local inflammation and osteoclast-mediated bone destruction in periodontitis. FASEB J..

[56] Oh D.Y., Talukdar S., Bae E.J., Imamura T., Morinaga H., Fan W., Li P., Lu W.J., Watkins S.M., Olefsky J.M. (2010). Gpr120 is an ω-3 fatty acid receptor mediating potent anti-inflammatory and insulin-sensitizing effects. Cell.

[57] Itariu B.K., Zeyda M., Hochbrugger E.E., Neuhofer A., Prager G., Schindler K., Bohdjalian A., Mascher D., Vangala S., Schranz M. (2012). Long-chain n-3 pufas reduce adipose tissue and systemic inflammation in severely obese nondiabetic patients: A randomized controlled trial. Am. J. Clin. Nutr..

[58] Elabdeen H.R., Mustafa M., Szklenar M., Ruhl R., Ali R., Bolstad A.I. (2013). Ratio of pro-resolving and pro-inflammatory lipid mediator precursors as potential markers for aggressive periodontitis. PLoS ONE.

[59] Leigh N.J., Nelson J.W., Mellas R.E., Aguirre A., Baker O.J. (2014). Expression of resolvin d1 biosynthetic pathways in salivary epithelium. J. Dent. Res..

[60] Azuma M., Aota K., Tamatani T., Motegi K., Yamashita T., Harada K., Hayashi Y., Sato M. (2000). Suppression of tumor necrosis factor α-induced matrix metalloproteinase 9 production by the introduction of a super-repressor form of inhibitor of nuclear factor kappabalpha complementary DNA into immortalized human salivary gland acinar cells. Prevention of the destruction of the acinar structure in Sjogren’s syndrome salivary glands. Arthritis Rheum..

[61] Nelson J.W., Leigh N.J., Mellas R.E., McCall A.D., Aguirre A., Baker O.J. (2014). Alx/FPR2 receptor for RVD1 is expressed and functional in salivary glands. Am. J. Physiol. Cell Physiol..

[62] Odusanwo O., Chinthamani S., McCall A., Duffey M.E., Baker O.J. (2012). Resolvin d1 prevents TNF-α-mediated disruption of salivary epithelial formation. Am. J. Physiol. Cell Physiol..

